# The Astrocyte-Targeted Therapy by Bushi for the Neuropathic Pain in Mice

**DOI:** 10.1371/journal.pone.0023510

**Published:** 2011-08-18

**Authors:** Keisuke Shibata, Takeshi Sugawara, Kayoko Fujishita, Youichi Shinozaki, Takashi Matsukawa, Tsutomu Suzuki, Schuichi Koizumi

**Affiliations:** 1 Department of Neuropharmacology, Interdisciplinary Graduate School of Medicine and Engineering, University of Yamanashi, Yamanashi, Japan; 2 Kenyudo Clinic, Yamanashi, Japan; 3 Department of Anesthesiology, Interdisciplinary Graduate School of Medicine and Engineering, University of Yamanashi, Yamanashi, Japan; 4 Department of Toxicology, School of Pharmacy and Pharmaceutical Sciences, Hoshi University, Tokyo, Japan; Okayama University Graduate School of Medicine, Dentistry and Pharmaceutical Sciences, Japan

## Abstract

**Background:**

There is accumulating evidence that the activation of spinal glial cells, especially microglia, is a key event in the pathogenesis of neuropathic pain. However, the inhibition of microglial activation is often ineffective, especially for long-lasting persistent neuropathic pain. So far, neuropathic pain remains largely intractable and a new therapeutic strategy for the pain is still required.

**Methods/Principal Findings:**

Using Seltzer model mice, we investigated the temporal aspect of two types of neuropathic pain behaviors, i.e., thermal hyperalgesia and mechanical allodynia, as well as that of morphological changes in spinal microglia and astrocytes by immunohistochemical studies. Firstly, we analyzed the pattern of progression in the pain behaviors, and found that the pain consisted of an “early induction phase” and subsequent “late maintenance phase”. We next analyzed the temporal changes in spinal glial cells, and found that the induction and the maintenance phase of pain were associated with the activation of microglia and astrocytes, respectively. When Bushi, a Japanese herbal medicine often used for several types of persistent pain, was administered chronically, it inhibited the maintenance phase of pain without affecting the induction phase, which was in accordance with the inhibition of astrocytic activation in the spinal cord. These analgesic effects and the inhibition of astrocytic activation by Bushi were mimicked by the intrathecal injection of fluorocitrate, an inhibitor of astrocytic activation. Finally, we tested the direct effect of Bushi on astrocytic activation, and found that Bushi suppressed the IL-1β- or IL-18-evoked ERK1/2-phosphorylation in cultured astrocytes but not the ATP-evoked p38- and ERK1/2-phosphorylation in microglia *in vitro*.

**Conclusions:**

Our results indicated that the activation of spinal astrocytes was responsible for the late maintenance phase of neuropathic pain in the Seltzer model mice and, therefore, the inhibition of astrocytic activation by Bushi could be a useful therapeutic strategy for treating neuropathic pain.

## Introduction

Neuropathic pain is the chronic pain state after a lesion or disease of the peripheral or central nervous system such as bone compression in cancer, diabetes, infection and acquired immunodeficiency syndrome [1]. A common symptom of this disease is mechanical allodynia, a painful response to innocuous tactile stimuli, and a decrease in nociceptive thresholds to the stimuli [2]. It has been reported that millions of people are suffering from neuropathic/chronic pain in the world [3], but most analgesics including non-steroidal anti-inflammatory drugs (NSAIDs) and even opioids, lack satisfactory efficacy for neuropathic pain [4], and they sometimes produce undesirable side effects [5].

There has been a paradigm shift in the understanding of the pathogenesis of neuropathic pain. Although neuropathic pain has long been considered to be caused by relevant changes in neurons, emerging lines of evidence have revealed that morphological and functional changes also occur in spinal glial cells [6], [7], [8], especially in microglia, the immune cells in the CNS [2], [3], [9], [10], [11], [12]. After peripheral nerve injury, microglia change their shapes and characteristic features from ‘resting’ to ‘activated’ through a series of cellular and molecular changes. Such activated microglia produce several diffusible molecules such as BDNF, by which microglia alter the excitability of adjacent neurons in the dorsal horn, leading to tactile allodynia [13]. Therefore, pharmacological and genetic manipulations of microglial functions could be a useful strategy for the control of neuropathic pain. Minocycline, an inhibitor of microglia, actually attenuates the development of neuropathic pain, but importantly, it often fails to suppress existing or established neuropathic pain [14], [15], suggesting a limited role of microglial activation in the neuropathic pain.

In addition to microglia, there is accumulating evidence that spinal astrocytes also change their morphology and function in the pathology of neuropathic pain, suggesting their involvement in neuropathic pain [16], [17]. Astrocytes directly alter neurotransmission because they encapsulate synapses and are in close contact with neuronal somas [18]. Importantly, changes in the astrocytic activities are known to dynamically alter the efficacy of neurotransmission in both physiological and pathological conditions [19], [20], [21], suggesting a possible linkage between astrocytic activation and neuropathic pain. In some cases, suppression of astrocytic activation by the astrocyte toxin, fluorocitrate, leads to an analgesic effect for neuropathic pain in several types of models [22], [23], [24]. Thus both microglia and astrocytes seem to be involved in neuropathic pain, but the temporal patterns of their activations are completely different, i.e., CD11b, a microglia specific complement receptor 3, is upregulated earlier whereas GFAP, an astrocyte specific intermediate filament, is upregulated later after the nerve injury [23]. Microglia upregulate interleukin (IL)-18 in the early induction phase, by which astrocytes are activated in the late maintenance phase of tactile allodynia [25]. After nerve injury, microglia are activated by MMP-9-cleaved IL-1β in the early pain phase, which is followed by astrocytic activation induced by MMP-2-cleaved IL-1β in the later phase [26]. Based on these reports, microglia and astrocytes are likely to have distinct roles in the pathology of neuropathic pain. However, little is known about differences in the temporal pattern of activation in each type of glial cell, or differences related to the progression or maintenance the neuropathic pain. In addition, so far, there is no available medicine can selectively inhibit each type of glial cells to reveal analgesic effect.

Although neuropathic pain is generally considered a refractory disease [4], some herbal medicines are known to be effective for such pain. Herbal medicines and acupuncture [27] have attracted worldwide attention for chronic pain relief. Japanese herbal medicine, known as Kampo, was originally based on Chinese herbal medicine, but has developed independently and is a standardized form of herbal medicine or remedy in terms of the quality and quantities of the ingredients. Similar to Western medicine, the quality and quantities of ingredients are strictly controlled, and many of them are often used for several types of diseases [28], [29]. Among herbal medicines, we focused on Bushi (TJ-3023, Tsumura, Japan), which is derived from aconite and is contained in several Kampo medicines such as Gosha-jinki-gan, Hachimi-jio-gan and Shinbu-to. They are known to be effective for streptozotocin-induced diabetic autonomic neuropathy [30], paclitaxel-induced peripheral neuropathy [31] and some other types of pain [32], [33]. In addition and more importantly, Bushi has been effective for several types of chronic and persistent pain including neuropathic pain and, is therefore, often used for such patients in our university hospital (Univ. Yamanashi Hospital, personal communication). Although the analgesic effect of herbal medicine is well documented, little is understood about its biological basis. Thus, elucidation of the molecular mechanism(s) underlying the analgesic effect of Bushi contributes to understanding the pathology of neuropathic pain and, in addition, it could provide important clues to a new strategy of medical treatment.

In the present study, we show that neuropathic pain consists of the microglia-mediated early induction phase and the subsequent astrocyte-mediated maintenance phase in Seltzer model mice, and demonstrate that Bushi exerts its analgesic effect against the maintenance phase of neuropathic pain by inhibiting the activation of astrocytes but not microglia.

## Results

### Effects of Bushi on pain behaviors and spinal glial activation in Seltzer model mice

**Figure 1:**
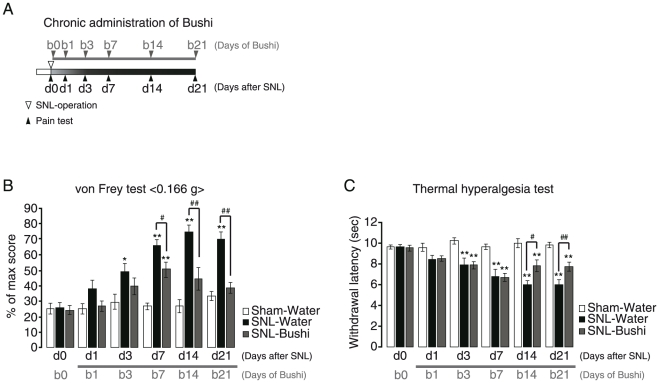
Analgesic effects of Bushi on pain behaviors in Seltzer model mice. A. Time schedule of chronic administration of Bushi. Either water or Bushi (300 mg/kg) administration (p.o., once-daily) was started right after SNL-operation. B, C. Pain behaviors induced by SNL, showing the effects of Bushi. SNL-Water developed mechanical allodynia (B) and thermal hyperalgesia (C); there was no change in either pain behaviors in Sham-Water. Bushi reduced both pain behaviors relatively late after the operation. d, days after SNL; b, days of Bushi administration. *p<0.05, **p<0.01 versus Sham-Water. #*p*<0.05, ##*p*<0.01 versus SNL-Water.

Throughout the experiments, we used Seltzer model mice [sciatic nerve ligation (SNL)-mice] as a neuropathic pain model. Bushi was orally administered to SNL-mice (SNL-Bushi) and the pain behaviors were assessed by both von Frey and thermal hyperalgesia tests for mechanical allodynia and thermal hyperalgesia, respectively. For comparison with Bushi-treated animals, water was orally administered to sham-operated and SNL-operated mice (i.e. Sham-Water and SNL-Water). Bushi (300 mg/kg) was administered to mice right after SNL operation and the pain behaviors were tested every day from 24 hr after the final administration of chemicals (Fig. 1A). The concentration of Bushi used in the present study was almost ED_50_ value. Chronic administration of Bushi had no effect on both von Frey test [23.3±1.9% (d0) v.s. 26.7±1.9% (d7), n = 3] and the thermal hyperalgesia test [9.9±0.1 sec (d0) v.s. 10.1±0.2 sec (d7), n = 3] in sham-operated mice. After SNL-operation, the pain behaviors in both tests gradually increased till 7 days after the operation (d7), when they were followed by constant and sustained pain behaviors (d14–d21) (Fig. 1, B and C). Therefore, in this study, we defined the former (i.e. 1–7 days after SNL operation) and the latter (i.e. >7 days after SNL operation) as ‘early induction’ and ‘late maintenance’ phases, respectively. In the Sham-Water group, no pain behavior was observed. In the von Frey test, significant pain behavior was observed at 3 days after the operation [25.5±3.3% (d0) v.s. 48.9±5.5% (d3), n = 9] and peaked at d14 (74.4±5.0%, n = 9). Similarly, in the thermal hyperalgesia test, the pain behavior became significant at 3 days after the operation [9.7±0.3 sec (d0) v.s. 7.9±0.6 sec (d3), n = 9] and peaked at d14 (6.1±0.3 sec). Bushi was effective on the late maintenance phase (i.e. from d7 to d21) of pain but appeared to be ineffective on the early induction phase (i.e. from d0 to d7) (Fig. 1, B and C).

**Figure 2:**
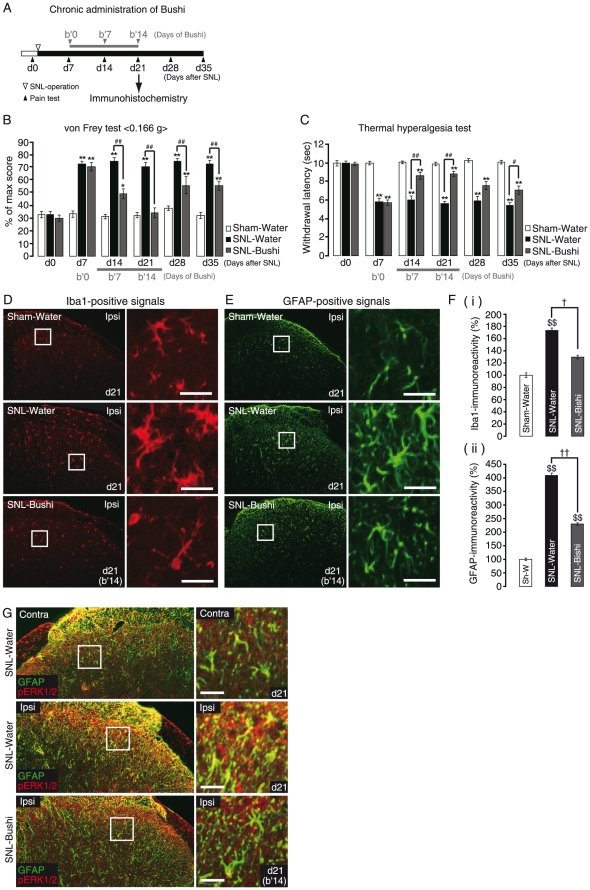
Effects of Bushi on the late maintenance phase of neuropathic pain. A. Time schedule of chronic administration of Bushi. Administration of water or Bushi (300 mg/kg) (p.o., once-daily) was started from d7 to d21 for 2 weeks, and withdrawn thereafter. B, C. Analgesic effect of Bushi on late maintenance phase of mechanical allodynia (B) and thermal hyperalgesia (C). At d7, both mechanical allodynia and thermal hyperalgesia were observed in SNL-Water and SNL-Bushi. These pain behaviors were maintained at least until d35 in SNL-Water. Bushi reduced the pain behaviors at d14/b′7 and d21/b′14, and even at d35 (2 weeks after withdrawal of Bushi). D, E. Immunohistochemical analysis of spinal microglia (D) and astrocytes (E). In SNL-Water, both Iba1- (upper panels) and GFAP- (lower panels) positive immunoreactivities were increased on d21 within the ipsilateral side of the dorsal horn (layer 1–2 area). A 2-week Bushi administration markedly suppressed both immunoreactivities. F. Semi-quantitative analysis of Iba1- (i) and GFAP- (ii) expression. Data indicate the relative mean immunofluorescence intensity of a single cell (n = 10). G. Double immunostaining of dorsal spinal cord with anti-pERK1/2 and -GFAP antibodies. In SNL-Water, pERK1/2-immunoreactivities (red) were increased in the ipsilateral side of the dorsal horn (d21), and were co-localized with the GFAP-signals (green). Bushi (2-week administration) also inhibited pERK1/2 in astrocytes. d, days after SNL; b″, days of Bushi administration. *p<0.05, **p<0.01 versus Sham-Water. #*p*<0.05, ##*p*<0.01 versus SNL-Water. $*p*<0.05, $$*p*<0.01 versus SNL-Water Contra. †*p*<0.05, ††*p*<0.01 versus SNL-Water Ipsi. Scale bars: 20 µm.

To examine whether Bushi is effective to only the late maintenance phase but not to the early induction phase of pain, the initiation of Bushi-administration was started 7 days after the SNL operation (Fig. 2A, from b′0 to b′14). At d7 (b′0), SNL-Water exhibited a dramatic increase in pain behaviors in both the von Frey test [33.0±2.5% (d0) v.s. 72.5±2.5% (d7), n = 12] (Fig. 2B) and the thermal hyperalgesia test [10.1±0.2 sec (d0) v.s. 5.8±0.4 sec (d7), n = 12] (Fig. 2C). These pain behaviors were maintained for at least 35 days after the operation (72.5±5.0% in the von Frey test and 5.4±0.5 sec in the thermal hyperalgesia test). Bushi revealed analgesic effects against the late phase of neuropathic pain (b′7) (49.5±7.0% and 8.7±0.5 sec in the von Frey and thermal hyperalgesia tests, respectively). A 14-day administration of Bushi (b′14) almost completely inhibited the pain behaviors (34.5±3.5% and 8.9±0.5 sec in the von Frey and thermal hyperalgesia tests, respectively). Thus, Bushi attenuated the established maintenance phase but not the early induction phase of neuropathic pain. Interestingly, even after Bushi withdrawal, the analgesic effects lasted for at least another 2 weeks, although they were slightly reduced [55.8±2.7% (d28) and 55.8±3.3% (d35) in the von Frey test; and 7.6±0.6 sec (d28) and 7.2±0.3 sec (d35) in the thermal hyperalgesia test].

We next observed the morphological changes in spinal microglia and astrocytes in the dorsal horn of the layer 1–2 at d21 in the late maintenance phase of neuropathic pain. It is well known that this spinal area is important for pain sensation because the primary afferent neurons form synapses to the secondary afferent ones, and thus both the activated microglia and astrocytes observed in this area after SNL operation could modulate the sensory transmission. In the Sham-Water group, microglia were ramified with long branching processes and small cellular bodies (Fig. 2D, upper panels, Sham-Water), and the astrocytes did not exhibit any sign of activation showing typical resting morphologies with thin processes and small cell bodies (Fig. 2E, top panels, Sham-Water). The morphologies of both microglia and astrocytes dramatically changed after the SNL operation, i.e., microglia showed ameboid shapes with short and thick processes (Fig. 2D, middle panels, SNL-Water), and astrocytes also appeared hypertrophic with thick processes (Fig. 2E, middle panels, SNL-Water). A 2-week administration of Bushi dramatically changed the glial morphologies into the resting ones (Fig. 2D and E, bottom panels, SNL-Bushi). We quantified both Iba1- and GFAP-immunoreactivities, showing the effect of Bushi. The intensity of the SNL-induced increase in both Iba1- and GFAP-positive signals was significantly decreased by 2-week administration of Bushi [Fig. 2F, Iba1- (i) and GFAP-immunoreactivities (ii)]. It is well known that, after nerve injury, extracellular signal-regulated kinase 1 and 2 (ERK1/2) are activated in spinal astrocytes, followed by the synthesis of several proinflammatory/nociceptive mediators, thereby leading to enhanced and prolonged pain [34]. In fact, the immunoreactivity of phosphorylated-ERK1/2 (pERK1/2), a member of mitogen-activated protein kinases (MAPKs) was increased in GFAP-positive astrocytes in our models (Fig. 2G, SNL-Water, middle panels). The administration of Bushi dramatically decreased the pERK1/2 signals in astrocytes (Fig. 2G, bottom panels).

**Figure 3:**
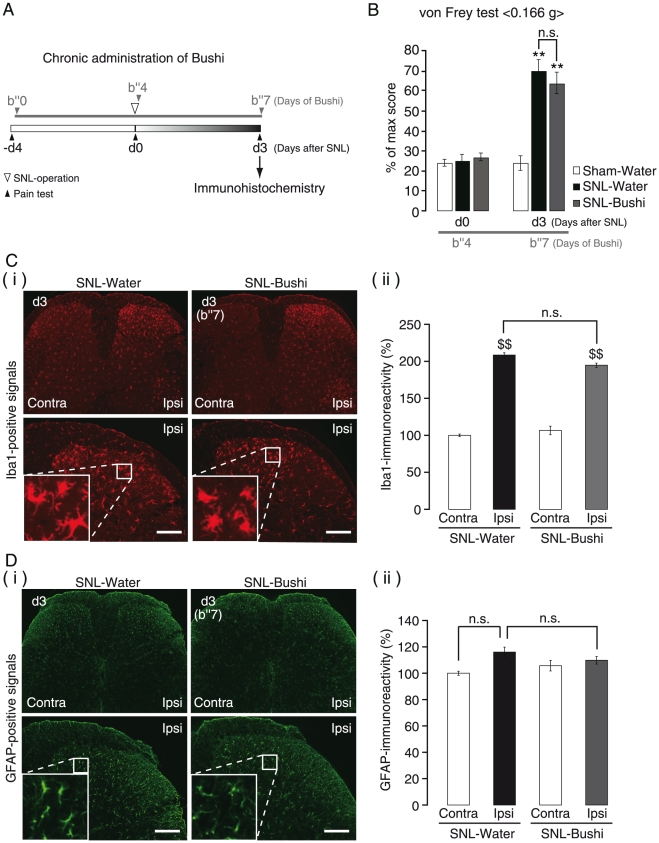
Effects of Bushi on the induction phase (early phase) of neuropathic pain. A. Time schedule of chronic administration of Bushi. Either water or Bushi (300 mg/kg) administration (p.o., once-daily) was started from −d4 to d3. B. Mechanical allodynia induced by SNL at d3. A 7-day administration of Bushi was not effective on the mechanical allodynia at d3/b″7. C, D. Effects of Bushi on the morphological change of spinal microglia (C) and astrocytes (D) in the early induction phase (i). Semi-quantitative analysis of Iba1- and GFAP-expression (ii). Data indicate the relative mean immunofluorescence intensity of a single cell (n = 10). Bushi did not suppress the increase in Iba1-immunoreactivity by SNL within the ipsilateral side of the dorsal horn (i.e. layer 1–2 area) at d3 (C). The elevation of GFAP-immunoreactivity had not occurred at d3 (D). d, days after SNL; b′, days of Bushi administration. **p<0.01 versus Sham-Water. $$*p*<0.01 versus SNL-Water Contra. Scale bars: 100 µm.

To confirm that Bushi was not effective in the early induction phase, it was administered to mice only in the early induction phase. For this experiment, the administration of Bushi was initiated 4 days before the SNL operation (Fig. 3A). Although 7-day administration of Bushi was enough to reduce the pain behaviors in the late maintenance phase (Fig. 2, B and C), administration for 7 days or longer in the early induction phase showed no analgesic effect [von Frey test; 24.0±3.7% (Sham-Water), 70.0±5.7% (SNL-Water), and 64.0±5.3% (SNL-Bushi), n = 5] (Fig. 3B). Three days after SNL (d3), microglia showed activated shapes, whereas the astrocytes looked normal (Fig. 3, C-(i) and D-(i), SNL-Water). Unlike its effect on astrocytes, a 7-day administration of Bushi (d3/b″7) failed to inhibit microglial activation (Fig. 3C-(i), SNL-Bushi). The significant augmentation of Iba1-immunoreactivity in the ipsi-lateral side by SNL operation remained at the same level at d3/b″7 [Fig. 3C-(ii)]. We could not observe any augmentation of GFAP-immunoreactivity by SNL operation at d3 [Fig. 3D-(ii)].

### Temporal relationship between spinal glial activation and pain behaviors in neuropathic pain

**Figure 4:**
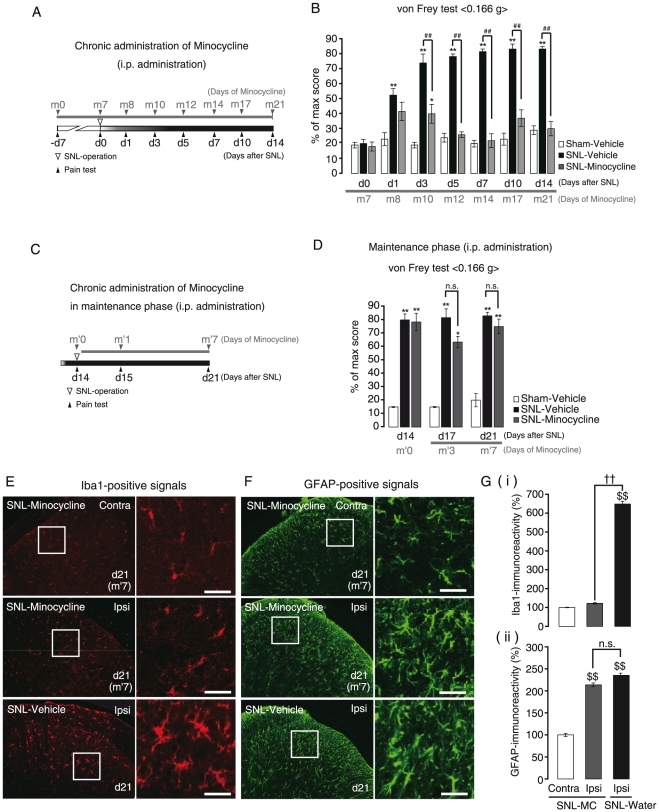
Role of microglial activation at the early induction phase and late maintenance phase of neuropathic pain. A. Time schedule of chronic administration of minocycline from the early induction phase to the late maintenance phase. Pain behavior was assessed by von Frey test at the time points indicated by arrowheads. B. Mechanical allodynia increased with time up to d7 and maintained thereafter in SNL-Water. Minocycline reduced the pain behavior both at the induction and maintenance phases. C. Time schedule of chronic administration of minocycline at the late maintenance phase. D. At the late maintenance phase, the established neuropathic pain by SNL was not decreased by minocycline. E, F. Morphological change of Iba1-positive spinal microglia (E) and augmentation of Iba1-immunoreactivity [G-(i)] by SNL were controlled by minocycline at the late maintenance phase. Minocycline had no effect on the morphological changes of GFAP-positive spinal astrocytes (F) and GFAP-immunoreactivity [G-(ii)]. d, days after SNL; m and m′, days of minocycline administration. *p<0.05, **p<0.01 versus Sham-Vehicle. ##p<0.01 versus SNL-Vehicle. $$*p*<0.01 versus SNL-MC (minocycline) Contra. ††*p*<0.01 versus SNL-MC Ipsi. Scale bars: 20 µm.

**Figure 5:**
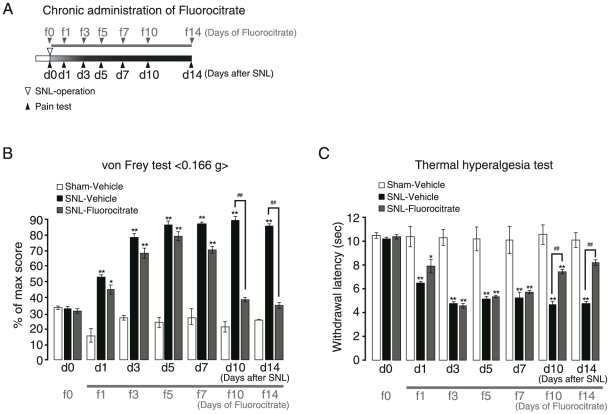
Effect of fluorocitrate on pain behaviors in the early induction phase and the late maintenance phase of neuropathic pain. A. Time schedule of chronic administration of fluorocitrate. Either vehicle or fluorocitrate (100 fmol/mouse) administration (i.t., once-daily) was started right after the SNL operation. B, C. Mechanical allodynia and thermal hyperalgesia were assessed from the early induction phase to the late maintenance phase. SNL-Vehicle developed mechanical allodynia (B) and thermal hyperalgesia (C); there was no change in the pain behaviors in Sham-Vehicle. Fluorocitrate reduced both pain behaviors only at the late maintenance phase, but was not effective at the early induction phase, as was the case with Bushi. d, days after SNL; f, days of fluorocitrate administration. *p<0.05, **p<0.01 versus Sham-Vehicle. ##*p*<0.01 versus SNL-Vehicle. Scale bars: 20 µm.

**Figure 6:**
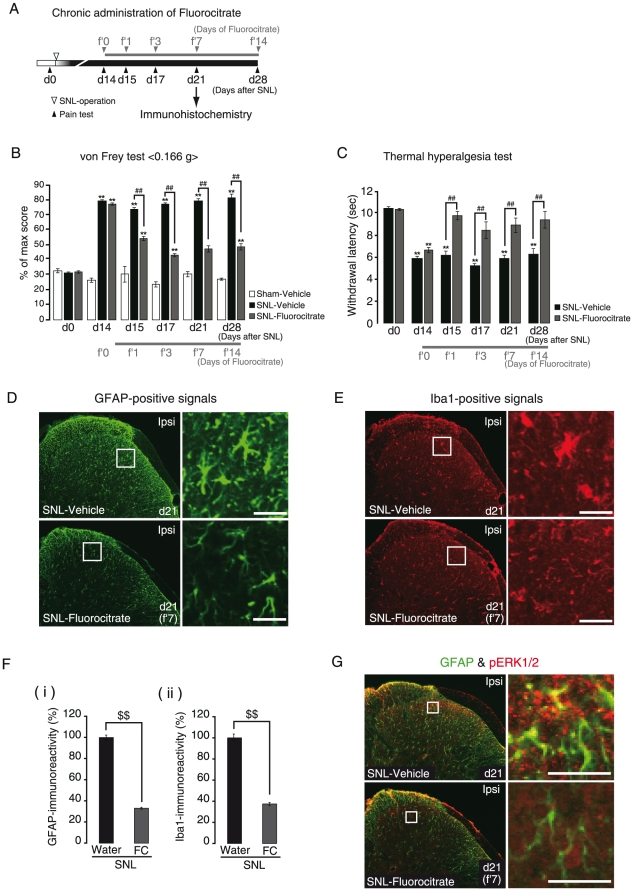
Role of astrocytic activation at the early induction phase and late maintenance phase of neuropathic pain. A. Time schedule of chronic administration of fluorocitrate, evaluating its effect on the late maintenance phase of neuropathic pain. Vehicle and fluorocitrate administration was started from d14 to d28 for 2 weeks. B, C. Analgesic effects of fluorocitrate on mechanical allodynia (B) and thermal hyperalgesia (C) in the late maintenance phase. Fluorocitrate revealed analgesic effects when it was administered in the late maintenance phase (from d15/f′1 to d28/f′14). D–F. Immunohistochemical analysis of spinal glial cells, showing the effects of fluorocitrate. Fluorocitrate reduced the morphological changes of GFAP-positive spinal astrocytes (D, lower panels) at d21/f′7 and the augmentation of GFAP- immunoreactivity [F-(i)], which was associated with the inhibition of pERK1/2 (G, lower panels). Fluorocitrate also reduced the morphological changes of Iba1-positive spinal microglia (E, lower pannels) and augmentation of Iba1-immunoreactivity [F-(ii)] at d21/f′7, as did Bushi. d, days after SNL; f′, days of fluorocitrate administration. **p<0.01 versus Sham-Vehicle. ##*p*<0.01 versus SNL-Vehicle. $$*p*<0.01 SNL-Water Ipsi versus SNL-FC (fluorocitrate) Ipsi. Scale bars: 20 µm.

To assess the temporal characteristic differences in the glial contribution to the neuropathic pain, we pharmacologically inhibited the activation of either microglia or astrocytes. The pain behaviors were tested every day from 24 hr after the final administration of chemicals. First, we selectively inhibited microglial activation by intraperitoneal (i.p.) administration of minocycline in SNL-mice. When minocycline administration was initiated from 7 days before SNL-operation (−d7/m0) to 14 days after SNL-operation (d14/m21) (Fig. 4A), the pain behaviors both at the early induction and at the late maintenance phases were significantly reduced (Fig. 4B and Table 1). We then investigated the effect of minocycline on the activation of spinal glial cells, and found that in this administration protocol, activation of astrocytes as well as microglia at the late maintenance phase (d14/m21) was inhibited (Fig. S1). However, when its administration was initiated from the late maintenance phase [from 14 days after SNL-operation (d14/m′0) to 21 days after SNL-operation (d21/m′7)] (Fig. 4C), it no longer showed any analgesic effects (Fig. 4D, Table 2). We again investigated the spinal glial morphology, and found that in this administration protocol, unlike the behavioral data, the administration of minocycline strongly inhibited the activation of microglia at the late maintenance phase (d21/m′7), i.e., the ameboid shape was reversed to the ramified one, and Iba1-immunoreactivity was decreased [Fig. 4, E, middle panels, and G-(i), SNL-Minocycline/Ipsi]. It should be noted that even when the microglial activation was reduced by minocycline, astrocytic activation persisted in the late maintenance phase [Fig. 4, F, middle panels, and G-(ii), SNL-Minocycline/Ipsi]. Next, we inhibited the astrocytic activation by fluorocitrate, a well-known metabolic inhibitor of astrocytes [23]. Fluorocitrate was administered intrathecally every day from right after the SNL operation (Fig. 5A). Fluorocitrate had no effect on the early induction phase of pain (Fig. 5, B, C, d1–d7/f1–f7 and Table 3), but significantly suppressed the late maintenance phase of pain (Fig. 5, B, C, d10–d14/f10–f14 and Table 3). Importantly, fluorocitrate showed a significant analgesic effect even when its administration was initiated 14 days after the SNL operation (d14/f′0), i.e., at the late maintenance phase (Fig. 6, A–C, and Table 4). In addition, fluorocitrate reversed the astrocytic activation, in association with a decrease in the GFAP-immunoreactivity [Fig. 6, D and F-(i)] and phosphorylation of ERK1/2 [Fig. 6G] at d21/f′7 (administered for 7 days from d14 to d21). It should be noted that fluorocitrate had no effect on the activation of microglia in the early induction phase, but did inhibit the activation of microglia in the late maintenance phase, as did Bushi [Fig. 6, E, and F-(ii)] [23].

**Table 1:** Effect of intraperitoneal administration of minocycline on mechanical allodynia (Fig. 4B).

von Frey test <0.166 g>						% of max score (%)	
	d0 (m7)	d1 (m8)	d3 (m10)	d5 (m12)	d7 (m14)	d10 (m17)	d14 (m21)
Sham-Vehicle	19.0±1.8	23.0±4.4	19.0±1.9	24.0±2.9	20.0±2.2	23.0±4.1	29.0±2.9
SNL-Vehicle	20.0±2.9	52.5±4.4**	74.0±6.0**	78.3±1.7**	81.7±1.7**	83.3±3.3**	83.3±1.7**
SNL-Minocycline	18.1±3.0	41.5±6.1	40.0±6.3* ^,^ ##	26.0±1.9##	22.0±4.6##	37.0±5.6##	30.0±4.7##

To assess mechanical allodynia, von Frey test was performed. The number of paw withdrawal times in response to a 0.166 g von Frey filament was counted and scored as followed; One trial involved 10 applications of filaments every 3 or 4 s, each of which was scored as 0, 1 or 2 as described in the methods section. The trial was evaluated based on a total score of 0–20 at the culmination. % of max score = (total score/2 10 trials) 100. Data show mean ± s.e.m. (n = 5–10).

*p<0.05,

**p<0.01 versus Sham-Vehicle,

## p<0.01 versus SNL-Vehicle.

d, days after SNL-operation; m, days of minocycline administration.

**Table 2:** Effect of intraperitoneal administration of minocycline on mechanical allodynia in the maintenance phase (Fig. 2D).

von Frey test <0.166 g>		% of max score (%)	
	d14 (m′0)	d17 (m′3)	d21 (m′7)
Sham-Vehicle	15.0±0.0	16.7±1.2	20.0±2.0
SNL-Vehicle	80.0±4.1	81.7±6.6**	83.0±2.3**
SNL-Minocycline	78.3±6.5	63.3±4.2*	75.3±5.4**

To assess mechanical allodynia, von Frey test was performed as described in table 1. Data show mean ± s.e.m. (n = 3).

*p<0.05,

**p<0.01 versus Sham-Vehicle.

d, days after SNL operation; m′, days of minocycline administration.

**Table 3:** Effect of intrathecal administration of fluorocitrate on mechanical allodynia and thermal hyperalgesia in the induction phase (Fig. 5, B and C).

von Frey test <0.166 g>						% of max score (%)	
	d0 (f0)	d1 (f1)	d3 (f3)	d5 (f5)	d7 (f7)	d10 (f10)	d14 (f14)
Sham-Vehicle	33.3±1.2	15.0±4.1	26.7±1.2	23.3±3.1	26.7±5.1	21.7±3.1	25.0±0.0
SNL-Vehicle	32.0±1.7	53.0±1.7**	78.0±2.9**	86.0±2.4**	87.0±1.3**	89.0±2.4**	85.0±1.4**
SNL-Fluorocitrate	31.0±1.5	45.0±3.1*	68.0±3.6**	79.0±2.7**	70.0±2.6**	39.0±1.5##	35.0±1.8##

Thermal hyperalgesia test						Withdrawal latensy (sec)	
	d0 (f0)	d1 (f1)	d3 (f3)	d5 (f5)	d7 (f7)	d10 (f10)	d14 (f14)
Sham-Vehicle	10.5±0.9	10.4±0.2	10.2±0.2	10.2±1.0	10.0±1.1	10.6±0.8	10.1±0.6
SNL-Vehicle	10.2±0.1	6.5±0.1**	4.8±0.1**	5.1±0.2**	5.2±0.4**	4.7±0.2**	4.8±0.2**
SNL-Fluorocitrate	10.4±0.2	7.9±0.6*	4.5±0.2**	5.3±0.1**	5.7±0.2**	7.4±0.2** ^,^ ##	8.2±0.3##

For mechanical allodynia, von Frey test was performed as described in table 1. Thermal hyperalgesia test was evaluated by the latency of paw withdrawal after the thermal stimulus. The value was determined as the average of four measurements per paw in thermal hyperalgesia test. Data show mean ± s.e.m. (n = 5).

*p<0.05,

**p<0.01 versus Sham-Vehicle,

## p<0.01 versus SNL-vehicle.

d, days after SNL operation; f, days of fluorocitrate administration.

**Table 4:** Effect of intrathecal administration of fluorocitrate on mechanical allodynia and thermal hyperalgesia in the maintenance phase (Fig. 6, B and C).

von Frey test <0.166 g>					% of max score (%)	
	d0	d14 (f′0)	d15 (f′1)	d17 (f′3)	d21 (f′7)	d28 (f′14)
Sham-Vehicle	33.3±1.4	23.1±4.1	30.0±5.0	21.3±1.1	26.7±1.4	25.0±0.6
SNL-Vehicle	31.6±0.4	79.7±0.6**	73.9±1.4**	78.1±0.6**	81.7±1.0**	83.3±1.6**
SNL-Fluorocitrate	32.2±0.5	76.9±0.6**	54.5±1.2** ^,^ ##	41.6±0.7**	45.4±1.5##	42.9±2.1** ^,^ ##

Thermal hyperalgesia test					Withdrawal latensy (sec)	
	d0	d14 (f′0)	d15 (f′1)	d17 (f′3)	d21 (f′7)	d28 (f′14)
SNL-Vehicle	10.5±0.1	5.8±0.1**	6.2±0.4**	5.3±0.2**	5.8±0.2**	6.3±0.5**
SNL-Fluorocitrate	10.3±0.1	6.7±0.6**	9.7±0.4##	8.5±0.7##	8.9±0.7##	9.4±0.8##

For mechanical allodynia, von Frey test was performed as described in table 1. Thermal hyperalgesia test was evaluated as described in table 3. Data show mean ± s.e.m. (n = 7–14).

**p<0.01 versus Sham-Vehicle,

## p<0.01 versus SNL-Vehicle.

d, days after SNL operation; f′, days of fluorocitrate administration.

### Direct effects of Bushi on cultured astrocytes and microglia

**Figure 7:**
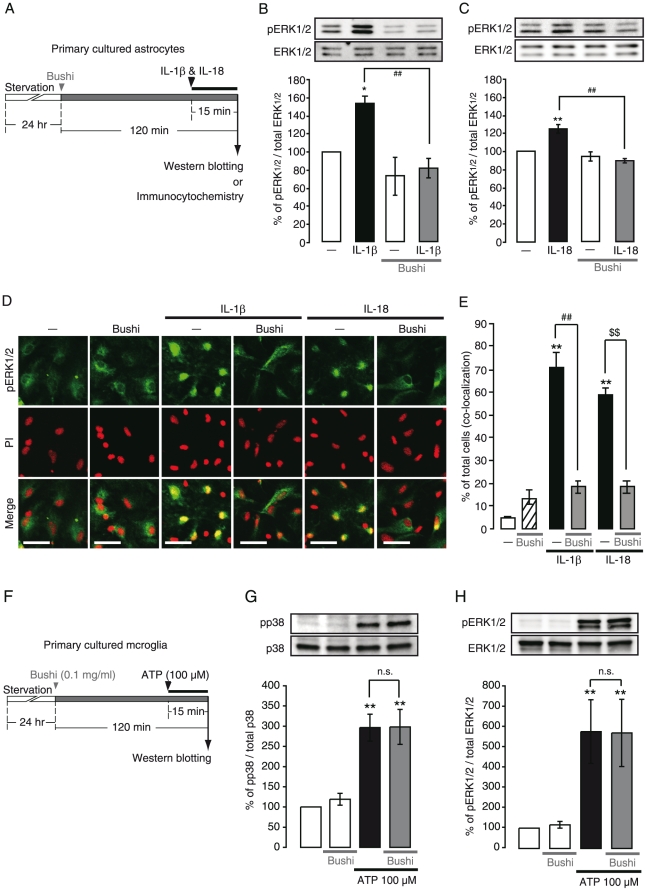
Direct effects of Bushi on activation in primary cultured astrocytes and microglia. A. Time schedule of administration of Bushi in primary cultured spinal astrocytes *in vitro*. Astrocytes were treated with Bushi (0.1 mg/ml) 105 min before and during IL-1β (10 ng/ml)- or IL-18 (10 ng/ml)-stimulation. B, C. The effect of Bushi on IL-1β- (B) or IL-18- (C) evoked ERK1/2 phosphorylation (pERK1/2). Bushi strongly inhibited pERK1/2 in astrocytes evoked by IL-1β or IL-18. D, E. The effect of Bushi on IL-1β- or IL-18- evoked pERK1/2 translocation. Bushi abolished the colocalization of pERK1/2 and PI signals evoked by IL-1β or IL-18 in cultured astrocytes. F. Time schedule of administration of Bushi in primary cultured cortical microglia *in vitro*. Microglia were treated with Bushi 105 min before and during ATP (100 µM)- stimulation. G, H. The effect of Bushi on ATP-evoked p38 phosphorylation (pp38) (G) and pERK1/2 (H). Bushi had no effect on ATP-evoked induction of both pp38 and pERK1/2 in cultured microglia. *p<0.05, **p<0.01, versus control. ##*p*<0.01 versus IL-1β. $$*p*<0.01, versus IL-18. Scale bars: 50 µm.

Both IL-1β and IL-18 are well-known proinflammatory cytokines that activate astrocytes causing chronic pain [25], [35], [36]. They activate intracellular signaling molecules including ERK1/2 [26] in astrocytes. When ERK1/2 is activated, it is phosphorylated and translocated from the cytoplasm into the nucleus [37]. We analyzed the effect of Bushi on the spatiotemporal behaviors of pERK1/2 in cultured spinal astrocytes. Western blotting analysis showed that both IL-1β and IL-18 (10 ng/ml, 15 min) activated ERK1/2 (i.e. an increase in pERK1/2) (Fig. 7, B and C). The increases in pERK1/2 proteins by IL-1β or IL-18 were almost completely inhibited by Bushi (0.1 mg/ml, 105 min pretreatment) [(DMEM, 100%; IL-1β, 152.9±9.6%; Bushi, 73.2±20.8%; Bushi/IL-1β, 80.8±11.0%) and (DMEM, 100%; IL-18, 124.0±4.8%; Bushi, 93.6±5.1%; Bushi/IL-18, 88.9±2.3%)]. To analyze the spatial behaviors of pERK1/2, propidium iodide (PI), a fluorescent dye that binds to DNA, was used for visualizing the nucleus. Without any stimulation, only faint pERK1/2 signals were observed in the entire cytoplasmic region. When astrocytes were stimulated with IL-1β or IL-18 (10 ng/ml) for 15 min, strong pERK1/2 signals were co-localized with the PI signals, suggesting the translocation of pERK1/2 into the nucleus (Fig. 7D). A 2-hr treatment of Bushi (0.1 mg/ml) dramatically inhibited the translocation of pERK1/2 (DMEM, 4.6±0.5%, n = 8 wells; Bushi, 13.4±2.8%, n = 9 wells; IL-1β, 70.8±5.7%, n = 9 wells; Bushi/IL-1β, 18.4±2.6%, n = 9 wells; IL-18, 58.7±2.5%, n = 8 wells; Bushi+IL-18, 18.4±2.6%, n = 8 wells) (Fig. 7E). Bushi did not inhibit the ATP-induced phosphorylation of p38 and ERK1/2 in primary cultured cortical microglia (Fig. 7, F–H).

## Discussion

In the present study, we demonstrated that Bushi inhibited the late maintenance phase of neuropathic pain without affecting the early induction phase of pain, in association with the suppression of astrocytic activation in the spinal cord. Although there is accumulating evidence that the activation of both microglia and astrocytes in the spinal cord is one of the key causes of neuropathic pain [6], [7], [8], astrocytes have received only limited attention in relation to pain. Here, however, we showed that the activation of spinal astrocytes has a more important role in the maintenance phase of neuropathic pain, and in addition and more importantly, we found that Bushi inhibits the activation of astrocytes to revealing analgesic effects against the chronic phase of neuropathic pain. Many neuropathic pain patients suffer from sustained and chronic pain for a long time and, therefore, it is of great importance that Bushi inhibits the maintenance phase of pain.

A recent breakthrough in pain research is the discovery of microglial involvement in the pathogenesis of neuropathic pain [9]. Since this report, many investigators have focused on microglia and made additional findings concerning the microglia-related mechanisms of pain pathology. After peripheral nerve injury, spinal microglia change their characteristic features and increase various chemicals such as neuropeptides, cytokines, chemokines, neurotransmitters, nucleotides and various receptors such as P2X_4_ receptors, which in turn, lead to sustained changes in the properties of the dorsal horn environment and the pain sensation [3], [9], [38]. Among them, much of the information transfer between activated microglia and neurons would be mediated by microglia-derived brain derived neurotrophic factor (BDNF) [13]. Microglia release BDNF in response to ATP via the activation of P2X_4_ receptors, which causes central sensitization by attenuating inhibitory synaptic transmission, leading to the onset of neuropathic pain. Thus, microglia are considered to be a potential target for neuropathic pain therapy as well as for understanding its pathogenesis. In the present Seltzer model mice, the time-course of the pain behaviors consisted of two phases, i.e., the early induction phase (<7 days after SNL) and the sustained maintenance phase (>7 days after SNL). Microglia had a pivotal role in the initiation of neuropathic pain in the early induction phase, and when their initial activation was inhibited by minocycline, the pain behavior was attenuated (Fig. 4B and Table 1). However, the analgesic effect of minocycline is time-dependent, i.e., it showed analgesic effects when administered at the early induction phase. When administration of minocycline was started at the late maintenance phase, it no longer showed any analgesic effects (Fig. 4, C and D). It should be noted that, in this administration protocol, minocycline did inhibit microglial activation but did not attenuate the pain behaviors at the late maintenance phase [Fig. 4, E and G-(i)]. These findings strongly suggest that activation of spinal microglia is no longer responsible for the late maintenance phase of neuropathic pain and that factors other than microglial activation should be involved in the later stage of neuropathic pain in this model.

Unlike minocycline, Bushi did not attenuate the early induction phase but, instead, it dramatically inhibited the late maintenance phase of pain, which suggests that Bushi, acting on minocycline-insensitive molecules, exerted its analgesic effects presumably via microglia-independent mechanisms. Recently, it has been reported that the activation of astrocytes is also involved in neuropathic pain and, interestingly, its involvement seemed to contribute to rather the chronic phase of pain [39], [40]. Similar to these reports, our present results showed that the activations of microglia and astrocytes were mainly observed in the early induction phase (Fig. 3C) and the late maintenance phase of neuropathic pain (Fig. 2, E, F-(ii), G, 4, F, G-(ii), 6, D, F-(i) and G), respectively. Bushi did not inhibit the activation of microglia in the early induction phase (Fig. 3C) but inhibited that of astrocytes in the late maintenance phase, and exerted analgesic effect against the late maintenance phase of neuropathic pain (Fig. 2, B–E). Similarly, fluorocitrate, a selective inhibitor of astrocytic activation [23], exerted analgesic effects against the late maintenance phase but not the early induction phase (Fig. 5, B and C). These findings strongly suggest that the activation of astrocytes is responsible for the late maintenance phase of neuropathic pain and that Bushi acts on astrocytes to exert its analgesic effect. However, when chronic administration of minocycline was initiated at the early induction phase, it inhibited pain behaviors even at the late maintenance phase as well as those at the early induction phase. In this administration protocol, minocycline also inhibited activation of spinal astrocytes at the late maintenance phases (Fig. S1), suggesting that microglial activation at the early induction phase should be a trigger for subsequent activation of astrocytes in the late maintenance phase in this model. Thus, activation of astrocytes is still important event for the maintenance phase of pain.

Although Bushi had no effect on microglial activation in the early induction phase (Fig. 3C), it suppressed the activation in the late maintenance phase of pain (Fig. 2, D and F-(i)). This finding raises the possibility that Bushi might also act on microglia and inhibit thier activation to reveal analgesic effect. However, we also found that (1) Bushi did not inhibit the ATP-induced activation (phosphorylation of p38 and ERK1/2) in primary microglia *in vitro* (Fig. 7, F–H), (2) fluorocitrate, an astrocytic inhibitor, also reduced the activation of microglia in the late maintenance phase [Fig. 6, E and F-(ii)]. All these findings suggest that Bushi secondarily suppresses the activation of microglia via the deactivation of astrocytes in the late maintenance phase.

The analgesic effect of Bushi is very interesting because (1) it was effective on established neuropathic pain (Fig. 2, B and C), (2) its analgesic effect was observed even when it was withdrawn (Fig. 2, B and C). Astrocytes are highly plastic but their changes are often reversible. The sustained activation of astrocytes is blocked by inhibition of their activation-feedforward loop [26]. Thus, Bushi may regulate reversible activation, and control the deactivation of astrocytes, thereby leading to its analgesic effect. The “activation” of astrocytes is a very ambiguous word that involves many types of astrocytic events or conditions and therefore the deactivation of astrocytes would also involve the inhibition of many molecules in astrocytes. In general, the activation of astrocytes is defined immunohistochemically as hypertrophic morphology with thick processes and increased GFAP-positive signals. Although upregulated GFAP itself might affect the astrocytic function [41], it is believed that many other events seen in spinal nerve injury, including the phosphorylation of MAP kinases [34] and increases in pro-inflammatory cytokines and chemokines [42], [43], would be more important for the pain sensation [44]. So far, however, it has remained unknown which astrocytic molecules are responsible for the maintenance phase of pain, and which molecule(s) “Bushi” inhibits to reveal its analgesic effects. With regard to pain-related astrocytic molecules, it has been reported that astrocytes could directly modulate the neuronal activity by releasing inflammatory cytokines including IL-1β in a chronic pain state via N-methyl-D-aspartate (NMDA) receptor-mediated mechanisms [45], [46]. Thus, such astrocytic pro-inflammatory cytokines would be among the most probable molecules that induce the maintenance phase of pain. With regard to the target molecule(s) of Bushi, we showed that one of the ERK1/2-mediated signaling molecules was involved. The activation of ERK1/2 by SNL results in the synthesis of several proinflammatory/nociceptive mediators, thereby leading to enhanced and prolonged neuropathic pain [34], [47]. In the present study, the immunoreactivity of pERK1/2 was increased by SNL in GFAP-positive spinal astrocytes (Fig. 2G, SNL-Water, middle panels), and was reversed by the administration of Bushi (Fig. 2G, bottom panels). In addition, both the activation and translocation of pERK1/2 induced by either IL-1β or IL-18, both of which are released from microglia [25], [35], [36], were also reversed by Bushi in cultured astrocytes *in vitro* (Fig. 7, B–E). These results suggest that Bushi acts on astrocytes directly and suppresses astrocytic activation, presumably via the inhibition of ERK1/2-related signals.

The time-courses in analgesic effects of Bushi on von Frey and thermal hyperalgesia tests were different, and von Frey test looks more sensitive to Bushi (Fig. 1, B and C). Although both tests are well-used indexes for neuropathic pain, mechanisms underlying mechanical allodynia and thermal hyperalgesia are not necessarily same, and therefore, distinct molecules are involved in the pain behaviors. For example, NMDA receptor is more important to induce thermal hyperalgesia than mechanical allodynia because MK801, a blocker of NMDA receptors, is more effective to thermal hyperalgesia than allodynia [48]. Thus, molecules involved in mechanical allodynia might be more sensitive to Bushi, but further investigation is required to clarify this. In addition, we must await further study to identify the pain-related molecules that are activated and inhibited by SNL and Bushi in astrocytes, respectively. Judging from previous studies, multiple molecules in astrocytes appear to be involved in the pain and be the targets of Bushi [44]. If this is the case, the multi-target therapy by Bushi might be a useful clinical strategy for the persistent neuropathic pain. Although fluorocitrate also showed an analgesic effect against the late maintenance phase of pain, it should be noted that unlike fluorocitrate, Bushi is appropriate for human application because it has already been used clinically for several diseases, and has not been reported to cause serious side effects as flurocitrate and its related chemicals did in animal models of neuropathic pain [49], [50].

Taken together, we demonstrated that Bushi acts on spinal astrocytes to reverse their activation, thereby leading to inhibition of the persistent late maintenance phase of neuropathic pain. A minute investigation of the molecular mechanisms underlying the Bushi-induced deactivation of astrocytes as well as its analgesic effects would lead to better understanding of the molecular basis of persistent neuropathic pain.

## Materials and Methods

### Animals and surgery

All procedures were performed in accordance with the “Guiding Principles in the Care and Use of Animals in the Field of Physiologic Sciences” published by the Physiologic Society of Japan [51] and with the previous approval of the Animal Care Committee of Yamanashi University (Chuo, Yamanashi, Japan). Using 8 week-old male ICR mice (Japan SLC, Shizuoka, Japan), we produced a partial sciatic nerve injury model by tying a tight ligature with an 8–0 silk suture around approximately 1/3–1/2 the diameter of the sciatic nerve located on the right-hand side (ipsilateral side). This method is similar to the approach described in rats by [52] and in mice by [53]. In sham-operated mice, the nerve was exposed without ligation.

### Drug treatment

We used clinically available Bushi (TJ-3023, Tsumura Co. Ltd., Japan), a processed aconiti tuber whose quality is strictly controlled by a 3D-HPLC analysis. Bushi was suspended and diluted with water for oral administration (30 mg/ml, 300 mg/kg, approximately 0.3–0.4 ml/mouse). Fluorocitrate (Sigma-Aldrich), an inhibitor of astrocytic activation, was administered intrathecally (100 fmol/5 µl/mouse). This is within the safe range for the selective effect of fluorocitrate on astrocytes without neuronal damage [23]. Minocycline (Sigma-Aldrich), an inhibitor of microglial activation, was administered intraperitoneally (30 mg/kg, approximately 0.3–0.4 ml/mouse). The dosage of minocycline used in the present study was based on a previous report [23].

### Behavioral analysis

On each testing day, the mice were brought into the behavior room 1 hr before the test session to allow them to habituate to the environment. Thermal hyperalgesia was assessed sensitivity to thermal stimulation. The hind paws of the mice were tested individually using a radiant thermal stimulus apparatus (model 33, Analgesia Meter; IITC/Life Science Instruments, Woodland Hills, CA, USA). The latency of the paw withdrawal after the thermal stimulus was determined as the average of four measurements per paw. Each paw was measured alternately after more than 5 min. The intensity of the thermal stimulus was adjusted to achieve an average baseline paw withdrawal latency of approximately 9–11 sec in naive mice. Only quick hind paw movements (either with or without licking of hind paws) away from the stimulus were considered to be withdrawal responses. Mechanical allodynia was quantified by measuring the hind paw withdrawal response sensitivity using von Frey filaments. Von Frey filaments were applied to the plantar surface of the hind paw. The paw withdrawal in response to the tactile stimulus was evaluated by scoring as follows: 0, no response; 1, a withdrawal response away from the stimulus with slight flinching and/or licking; 2, an intense withdrawal response away from the stimulus with brisk flinching and/or licking [54]. One trial involved 10 applications of filaments every 3 or 4 sec, each of which was scored as 0, 1 or 2. The trial was evaluated based on a total score of 0–20 at the culmination (% of max score).

### Immunohistochemistry

After perfusion, the lumbar spinal cord was postfixed in 4% paraformaldehyde for 24 hr, and then permeated with 20% sucrose in 0.1 M PBS for 24 hr and 30% sucrose in 0.1 M PBS for 48 hr at 4°C. Lumbar spinal cord segments were frozen in an embedding compound (Sakura Finetek, Tokyo, Japan) on dry ice. Frozen spinal segments were cut with a cryostat (Leica CM 1100; Leica, Wetzlar, Germany) at a thickness of 30 µm and collected in PBS at 4°C to be processed immunohistochemically as free-floating sections. The sections were incubated overnight at 4°C with primary antibodies: mouse anti-GFAP (1∶2000; Millipore), rabbit anti-Iba1 (1∶2000; a kind gift from Dr. S. Kohsaka, National Institute of Neurosciences, Tokyo, Japan) and rabbit anti-pERK1/2 (1∶500; Cell Signaling Technology). The sections were washed six times with 0.01 M PBS (10 min each) and then incubated for 3 hr at room temperature with the secondary antibody: Alexa488- and Alexa546-conjugated mouse- and rabbit-IgGs (Invitrogen). Immunohistochemical images were obtained using a confocal laser microscope (Fluoview1000; Olympus, Tokyo, Japan) and digital images were captured with Fluoview1000 (Olympus). For the comparison of double-stained patterns, images were processed using Photoshop 5 (Adobe System, Mountain View, CA).

### Cell culture

The culture of spinal astrocytes and cortical microglia were prepared as described [19] with minor modifications. Spinal astrocytes and cortical microglia were dissected from neonatal Wistar rats. To purify astrocytes (GFAP-positive cells) from the mixture of spinal cord cultures, the cells were subjected to 24 hr of continuous shaking 10–14 days after plating to remove detached cells. For the Western blotting analysis, cells were seeded on 6-well cell culture plates (greiner bio-one, Tokyo, Japan) at a density of 1×10^5^ cells/well. For the immunocytochemistry, cells were seeded on poly-L-lysine and collagen-coated 8-well chamber at a density of 2.5×10^4^ cells/well.

### Western blotting analysis

After IL-1β or IL-18-stimulation, cells were lysed and the lysates were resolved with 10% SDS-PAGE gels and transferred to PVDF membranes. The membranes were blocked for 1 hr in Tris-buffered saline containing 0.1% Tween-20 (TBS/T) and 5% bovine serum albumin (BSA) at room temperature. Then the membranes were incubated with the primary antibody: rabbit anti-pERK1/2 (1∶5000; Cell Signaling Technology) and rabbit anti-pp38 (1∶5000; Cell Signaling Technology) diluted with Can Get Signal® solution 1 (TOYOBO), immunoreaction enhancer solution, overnight at 4°C. After three washes with TBS/T, the membranes were incubated with horseradish peroxidase-conjugated anti-rabbit antibody (1∶20000; Amersham Pharmacia Biotech Inc., Piscataway, NJ, USA) diluted with Can Get Signal® solution 2 (TOYOBO) for 1 hr at room temperature. The membranes were washed with TBS/T three times, and the proteins were visualized by chemiluminescence. To detect total ERK1/2 and p38, aliquots of the same sample were resolved with SDS-PAGE gels, transferred to PVDF membranes in the same conditions and exposed to rabbit anti-total ERK1/2 antibody (1∶5000; Cell Signaling Technology) and rabbit anti-total p38 antibody (1∶5000; Cell Signaling Technology), respectively. To quantify the intensity of the each bands, we used ImageJ (http://rsb.info.nih.gov/ij/). Each bands were selected by rectangular selection. Then, we selected *Analyze-Gels-Select First Lane* from the menu bar. The area corresponding to each band was measured using the *Wand (tracing) tool* from the tool bar.

### Immunocytochemistry

After each treatment, the cells were fixed for 30 min at room temperature in 99% methanol. The fixed cells were permeabilized with PBS containing 0.1% Triton X-100 for 15 min at room temperature and then incubated with the polyclonal rabbit anti-pERK1/2 antibody for 24 hr at 4°C. After washing, the cells were incubated with the appropriate secondary antibody conjugated to Alexa 488 and propidium iodide (PI), a DNA biding dye, for 1 hr at room temperature. The images were obtained using a confocal laser microscope and digital images were captured with Fluoview1000. For the comparison of double-stained patterns, images were processed using Photoshop 5.

### Statistical Analysis

All results were expressed as mean ± SEM, together with the number of animals or cultured-wells. All data were analyzed using analyses of variance (ANOVA) performed with Scheffe multiple comparison tests. A *p* value less than 0.05 was considered to be statistically significant.

## Supporting Information

Figure S1 Suppression of microglial activation by minocycline at the erarly induction phase resulted in inhibition of subsequent astrocytic activation at the late maintenance phase. Immunohistochemical analysis of microglia (Iba-1-positive signals) (A, B) and astrocytes (GFAP-positive signals) (C, D), showing effect of chronic administration of minocycline (30 mg/kg, p.i.). The administration protocol was same as Fig. 4A (from −d7 to d14). Rectangulars in A and C were expanded and shown as high magnification images in the corresponding lower pannels. Suppression of microglial activation (A) resulted in inhibition of subsequent astrocytic activation at the late maintenance phase (E; SNL-Minocycline (d14/m21), Ipsi), which was summarized in B (microglia) and D (astrocytes). $$p<0.01 versus Sham-Vehicle Contra. †† <0.01 versus Sham-Vehicle Ipsi. Scale bars: 50 µm.
(EPS)
